# Development of a Functional Loog‐Pang Starter Using Selected Yeast and Fungal Strains for Black Glutinous Rice Sato Fermentation

**DOI:** 10.1155/tswj/2063235

**Published:** 2026-05-28

**Authors:** Surachai Rattanasuk, Ketsarin Thinthapthai, Natthakon Khamthawi

**Affiliations:** ^1^ Department of Science and Technology, Faculty of Liberal Arts and Science, Roi Et Rajabhat University, Roi Et, Thailand

**Keywords:** alcohol tolerance, amylase activity, black glutinous rice Sato, fermentation starter (Loog-pang), yeast and fungal isolation

## Abstract

The production of Sato using black glutinous rice offers a valuable opportunity to enhance its overall quality by leveraging the unique nutritional profile, bioactive compounds, and distinct sensory attributes of pigmented rice varieties. This study aims to develop an effective Loog‐pang starter to produce black glutinous rice Sato. Yeast and fungal strains were isolated from Loog‐pang Sato and Loog‐pang Khao Mak samples collected from Roi Et and Yasothon provinces in Thailand. Fungal strains were selected based on their amylase production on soluble starch agar, while yeast strains were screened for their fermentation performance based on TDS reduction, ethanol production, and alcohol tolerance. The study successfully isolated eight fungal strains and 14 yeast strains. Fungal isolates FSA1 and FSA2 exhibited the highest amylase activity on starch agar after iodine treatment. Yeast isolate YKB1 demonstrated the highest alcohol tolerance when cultured in liquid media containing 10% and 15% alcohol. For Sato fermentation, this study utilized a ratio of fungal strain (FSA1 or FSA2) to yeast strain (YKB1). The highest alcohol production, at 16%, was achieved using Loog‐pang made with the combination of FSA1 and YKB1. The yeast DNA sequencing analysis of YKB1 revealed a 95.57% similarity to *Issatchenkia orientalis*. The findings from this study present significant potential for enhancing Sato production by isolating yeast and fungi, enabling local producers to scale up production, improve production, and expand markets both locally and internationally.

## 1. Introduction

The beverage is commonly referred to as “rice wine” in the West due to its alcohol content, which is comparable to that of wine [1]. In Thailand, a traditional fermented rice beverage called Sato is widely produced and consumed. Sato is made from fermented white glutinous rice combined with Loog‐pang (a fermentation starter). It is believed to offer various health benefits, including aiding digestion, providing antioxidants, exhibiting antidiabetic potential, stimulating blood circulation, supplying bioactive compounds, enhancing the immune system, providing anticancer properties, and reducing stress [2]. The preparation of rice wine follows a standardized process that includes the saccharification of steamed rice starch. This transformation is facilitated by fungal enzymes produced by fungi such as *Aspergillus* and *Rhizopus* under aerobic solid‐state fermentation conditions [3]. Subsequently, molds are combined with water and subjected to submerged alcoholic fermentation, driven by diverse yeast cultures [4]. Various yeast strains have been reported for ethanol fermentation in rice wine, including *Saccharomyces cerevisiae* [3, 5, 6], *Saccharomycopsis capsularis*, *Saccharomycopsis fibuligera*, *Pichia anomala*, *Pichia fabianii*, *Pichia guilliermondii*, *Candida tropicalis*, *Candida utilis*, *Candida krusei*, *Hanseniaspora*, *Metschnikowia*, and *Kluyveromyces* [7, 8]. An optimal concentration of these alcohols contributes to the aromatic profile, thereby enhancing the overall sensory experience and desirability of the wine [1,9].

The effectiveness of Loog‐pang as a fermentation starter depends on the functional roles of its microbial community. Amylolytic fungi are essential for hydrolyzing rice starch into fermentable sugars during the saccharification stage, directly influencing substrate availability for fermentation [10, 11]. In parallel, alcohol‐tolerant yeast plays a critical role in converting these sugars into ethanol while maintaining activity under increasing alcohol concentrations. Variations in these functional traits among indigenous microbial populations can lead to inconsistent fermentation performance, affecting ethanol yield and overall process reliability [12]. Therefore, the selection of strains with strong amylase activity and high alcohol tolerance is a key strategy for improving Loog‐pang standardization. Loog‐pang is prepared by combining rice flour with a selection of herbs and spices, along with a powdered inoculum sourced from a previous batch to enhance fermentation [13].

Pigmented varieties, including black, purple, red, and brown glutinous rice, are rich in phenolic compounds, anthocyanins, and antioxidants, which contribute to their well‐documented health benefits. These include antioxidant activity, bioactive properties, anticancer potential [14], anti‐inflammatory effects, antidiabetic activity, and cholesterol‐lowering properties. In addition to its nutritional value, the presence of key volatile compounds further underscores its potential as a functional food ingredient. Consequently, glutinous rice has garnered increasing attention as a valuable raw material for the development of commercial health foods and dietary supplements [2]. However, due to the reliance on these natural microorganisms, the quality of Sato can be inconsistent. This includes fluctuating alcohol content across different batches, as well as undesirable flavors, such as overly sweet or sour notes, which make the beverage less palatable.

The inherent variability of microbial strains in Loog‐pang presents significant challenges in standardizing the fermentation process and enhancing overall efficiency. This inconsistency complicates efforts to scale up production and achieve consistent quality control, as fluctuations in microbial composition can impact taste, aroma, and alcohol content. Addressing these microbial variations is essential for optimizing Sato production and ensuring a stable, high‐quality final product. Research has demonstrated that isolating and selecting specific yeast and fungal strains can improve fermentation predictability and enhance desirable characteristics. Therefore, this study aimed to isolate amylolytic fungi from dried starter Khao Mak and alcohol‐tolerant yeast from dried starter Sato in order to select functional strains for the development of a more standardized starter culture for black glutinous rice Sato fermentation. The ultimate objective is to facilitate the production of premium‐quality black glutinous rice wine with greater consistency and reliability.

## 2. Materials and Methods

### 2.1. Collection of Loog‐Pang Sato and Loog‐Pang Khao Mak Samples

Loog‐pang samples were collected from Chiang Kwan District, Roi Et Province, and Sai Mun District, Yasothon Province, Thailand. The samples were stored in sealed containers or plastic bags and kept at room temperature for further experimentation.

### 2.2. Fungal Isolation

Loog‐pang samples were utilized to ferment 50 g of steamed black glutinous rice at a concentration of 0.5%. Following a 72‐h fermentation period at 30°C, fungal mycelial growth was observed. The fermented rice grains were subsequently transferred to potato dextrose agar (PDA) and incubated at 30°C for 72 h. Colonies exhibiting distinct morphological characteristics were selected for further purification, and the fungal isolates were preserved under refrigeration for future research.

### 2.3. Yeast Isolation

For yeast isolation, fermented black glutinous rice, prepared using 0.5% Loog‐pang, was incubated for 72 h at 30°C. The resulting sweet liquid, known as “Nam Toi,” was utilized for yeast isolation. Nam Toi was spread‐plated on yeast extract peptone dextrose (YPD) agar and incubated at 30°C for 72 h. Yeast colonies were then purified by cross‐streaking on YPD agar, and the purified yeast isolates were preserved for subsequent experimentation.

### 2.4. Selection of Fungal and Yeast Strains

Fungal strains were selected for amylase production using a method adapted from [15]. Each fungal isolate was cultured on PDA, and a sterile 6‐mm cork borer was used to transfer a sample from the colony edge to soluble starch agar (SA) containing 1% starch. After incubation at 30°C for 72 h, 1% iodine solution was applied to detect clear zones, signifying amylase activity. The diameters of these clear zones were measured to evaluate the amylase activity of the isolates. Selected fungal strains were then inoculated into 40 g of steamed black glutinous rice, incubated at 37°C for 7 days, and stored at 4°C for future use in Loog‐pang production.

Yeast isolates were designated with the prefix Y, whereas fungal isolates were designated with the prefix F, followed by source‐specific codes and isolate numbers.

### 2.5. Yeast Selection

The fermentative ability of yeast strains for sugar metabolism and ethanol production was evaluated. The yeast isolates were introduced into sterile 10‐mL tubes containing a 20°Brix sucrose solution, supplemented with 0.05% (*w*/*v*) diammonium phosphate, each equipped with a Durham tube for gas collection. One milliliter of yeast culture was inoculated into each tube. Fermentation was conducted at 30°C and 37°C for 5 days without agitation. Fermentation was assessed by measuring the reduction in sugar content using a hand refractometer, while gas production was monitored as an indicator of metabolic activity.

### 2.6. Alcohol Tolerance Testing

To assess alcohol tolerance, yeast isolates were evaluated in a solution with an initial concentration of 15°Brix, supplemented with 0.05% diammonium phosphate (*w*/*v*). Ethanol was added to achieve final concentrations of 10% and 15%. The solutions were aliquoted into 10‐mL tubes, sterilized at 110°C for 15 min, and inoculated with 1 mL of yeast culture. Fermentation was conducted at 30°C and 37°C for 5 days. Alcohol tolerance was evaluated based on gas production observed in the Durham tube.

### 2.7. Preparation of Yeast and Fungi for Loog‐Pang Production

For yeast inoculum preparation, yeast strains were cultured on YPD agar for 48 h, then transferred to 250‐mL flasks containing sterile 50 mL of YPD broth and incubated at 37°C with shaking at 200 rpm for 48 h. The yeast concentration was adjusted to 10^8^ cells/mL using a hemacytometer before further use.

For fungal starter preparation (Tane‐koji), 25 g of black glutinous rice was washed, soaked overnight (12–20 h), and drained. The rice was sterilized at 121°C for 15 min, cooled, and inoculated with 0.2% (*w*/*w*) fungal spores. The mixture was incubated at 30°C for 5 days to produce fungal inoculum.

### 2.8. Laboratory‐Scale Loog‐Pang Sato Production

The prepared yeast inoculum and Tane‐koji were mixed according to the formula for making the fermentation starter. The ratio of herbs is shown in Table 1. The ingredients are thoroughly combined and shaped into round balls, approximately 3 cm in diameter and weighing about 6–7 g. The balls are placed in a well‐ventilated container and covered with 2–3 layers of muslin cloth to allow for proper air circulation and microbial growth. They are incubated at room temperature for 2 days, then dried at 40°C for 1 day. Once dried, the starter is stored in airtight plastic bags to prevent moisture, ensuring its preservation for future fermentation use.

**Table 1:** Herbal composition ratios for Loog‐pang production.

Herbal composition	Weight (g)
*Allium sativum* powder	1.5
*Zingiber officinale* powder	1.5
*Alpinia galanga* powder	1.5
*Glycyrrhiza glabra* powder	1.5
*Piper nigrum* powder	1.5
*Cinnamomum verum* powder	1.5
*Piper longum* powder	0.5
*Oryza sativa* var. *glutinosa* powder	100

### 2.9. Loog‐Pang Efficiency Determination

To evaluate the fermentation of Loog‐pang, 100 g of black glutinous rice were soaked overnight. After draining the rice, it was wrapped in muslin cloth and steamed for 40 min. The rice was then cooled and sprinkled with approximately 250 mL of water to separate the grains. Six grams of finely ground Loog‐pang powder were evenly sprinkled and mixed with the rice, which was then placed in a jar or container with a tight‐fitting lid. The jar was sealed and incubated at room temperature for 72 h. Afterward, 500 mL of distilled water was added to the jar, which was sealed tightly and incubated for an additional 5 days (samples were collected every day). The fermented mixture was then filtered using muslin cloth and transferred to a container, followed by the addition of 2.4 g/L of potassium metabisulfite (KMS). The container was tightly sealed and stored at 4°C for preservation.

### 2.10. Yeast Identification

The fungal and yeast strains exhibiting optimal characteristics for Loog‐pang production were submitted to Macrogen Company (Republic of Korea) for DNA sequencing. The obtained sequences were subsequently analyzed using the BLAST program by comparing them against the nucleotide database at NCBI.

### 2.11. Statistical Data Analysis

Statistical data analysis was performed using basic statistics, including mean ($\bar{x}$), percentage (%), and standard deviation (SD). Statistical differences were analyzed using analysis of variance (ANOVA), and mean differences were tested using Duncan’s new multiple range test (DMRT) at a 95% confidence level. The analysis was conducted using SPSS software, Version 21.

## 3. Results

### 3.1. Isolation of Fungi and Yeasts

Fungi and yeasts were isolated from Loog‐pang Sato collected from two locations: Chiang Kwan District, Roi Et Province, and Sai Mun District, Yasothon Province. Loog‐pang Khao Mak fermentation starters were collected from three locations: Mueang District and Chiang Kwan District in Roi Et Province and Sai Mun District in Yasothon Province. A total of 22 isolates were obtained, including eight fungal isolates and 14 yeast isolates (Table 2).

**Table 2:** Number of fungal and yeast isolates from fermentation starters (Loog‐pang).

Loog‐pang	Location	Isolate no.	Amount	
			Fungi	Yeast
Sato	Chiang Kwan District	YKA1, YKA2, YKA3, YKB1, YKB2, YKB3, YKB4, YKB5, FKA1, FKA2	2	8
	Sai Mun District	YSA1, YSA2, YSA3, YSB1, YSB2, YSB3, FSA1, FSA2	2	6
Khao Mak	Mueang District	FMA1	1	—
	Chiang Kwan District	FKKA1	1	—
	Sai Mun District	FKSA1, FKSA2	2	—

### 3.2. Selection of Fungi for Starch Degradation

**Figure 1:**
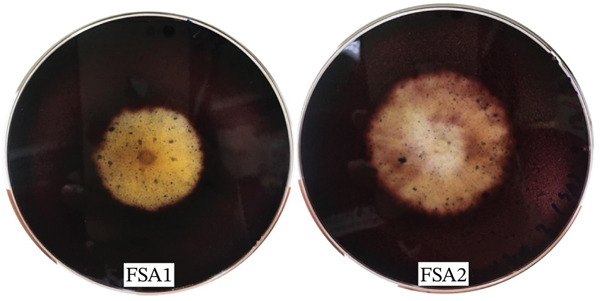
Starch‐degrading fungal isolates FSA1 and FSA2.

Selection of starch‐degrading fungi revealed two isolates, FSA1 and FSA2, exhibiting the highest starch degradation efficiency. The clear zone diameters of the starch‐degrading areas were measured at 3.6 and 2.9 cm, respectively (Figure 1).

### 3.3. Evaluation of Loog‐Pang Efficiency in Sugar Fermentation and Alcohol Tolerance

The study demonstrated that yeast isolates YKB4 and YKB1 exhibited significantly higher fermentation performance based on total dissolved solids (TDS) reduction compared to other isolates. When cultivated in a fermentation medium with an initial sugar concentration of 20°Brix, the sugar content decreased to 16.70 ± 0.20°Brix and 17.10 ± 0.20°Brix, respectively, after 5 days of fermentation. Furthermore, alcohol tolerance assays indicated that isolate YKB1 displayed superior resistance to alcohol concentrations of 10% and 15% compared to the other isolates. Notably, yeast isolate YKB1 exhibited the highest gas production and alcohol tolerance, highlighting its potential for industrial fermentation applications (Table 3).

**Table 3:** Efficiency of sugar fermentation and ethanol tolerance of isolated yeasts.

Isolate no.	Remaining sugar (^°^Brix)		10% alcohol		15% alcohol	
	30°C	37°C	30°C	37°C	30°C	37°C
YKA3	17.63^b^ ± 0.06	17.46^bc^ ± 0.15	+++	+	+	+
YKB5	17.40^a^ ± 0.10	17.63^c^ ± 0.25	+	+	+	+
YKB1	18.00^c^ ± 0.10	17.10^b^ ± 0.20	+++	+++	+	+
YKB4	17.64^b^ ± 0.09	16.70^a^ ± 0.20	+	+	+	+
YKA1	17.78^b^ ± 0.03	17.33^bc^ ± 0.25	++++	++	+	+

*Note:* Values with different letters in a column show significant differences (*p* ≤ 0.05). +: gas level at one‐quarter of the Durham tube; ++: gas level at one‐half of the Durham tube; +++: gas level at three‐quarters of the Durham tube; ++++: gas level at a full Durham tube.

### 3.4. The Characteristics of Black Glutinous Rice Wine

Through the selection process of fungal and yeast strains across various fermentation stages, the FKKA1 and FKSA1 fungal isolates exhibited the highest starch degradation efficiency, whereas the YKB1 yeast isolate demonstrated superior sugar‐to‐alcohol conversion efficiency and greater alcohol tolerance relative to other isolates. The optimal fungi‐to‐yeast ratio was determined to be 3:1, utilizing 100 g of black glutinous rice starch per sample, supplemented with 40 mL of distilled water and a specified mixture of herbs. This formulation resulted in the development of four distinct black glutinous rice fermentation starter cultures: FSA1:YKB1, FSA2:YKB1, FFA1:YKB1, and FSA1+FSA2:YKB1.

### 3.5. TDS

**Figure 2:**
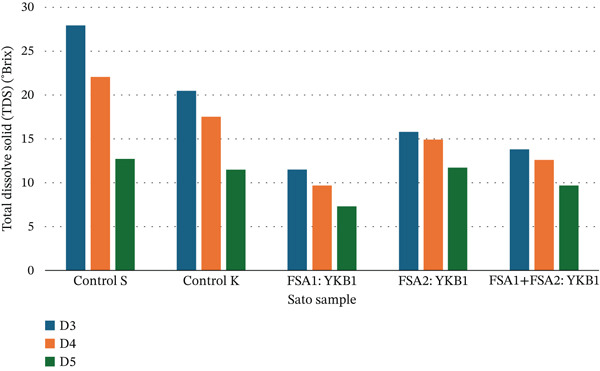
Total dissolved solids of six Sato samples.

An analysis of TDS dynamics during the 7‐day fermentation of black glutinous rice wine (Sato) indicated an initial increase in TDS content during the first 3 days across all six samples fermented with black glutinous rice starter cultures (Figure 2). Thereafter, TDS levels progressively declined throughout the remainder of the fermentation period. Among the six samples, Sample 3, fermented with a ratio of FSA1:YKB1, exhibited the most rapid sugar consumption, resulting in the lowest final TDS value of 7.3 ± 0.02°Brix.

### 3.6. pH Value

**Figure 3:**
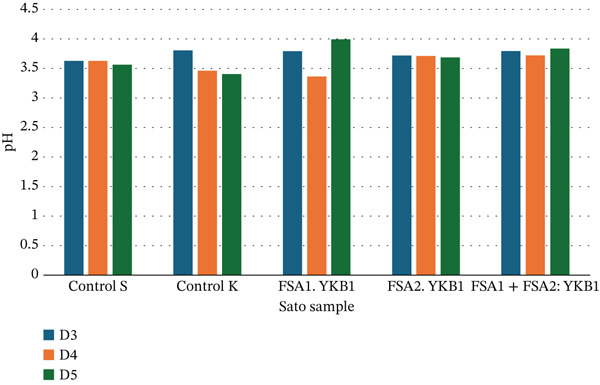
pH of Sato during fermentation.

The pH measurements during the 7‐day fermentation of Sato (Figure 3) indicated that the pH levels of Sato fermented with six different black glutinous rice starter cultures remained within the range of 3.40 ± 0.01 to 3.84 ± 0.01 throughout the fermentation process.

### 3.7. Ethanol Content

**Figure 4:**
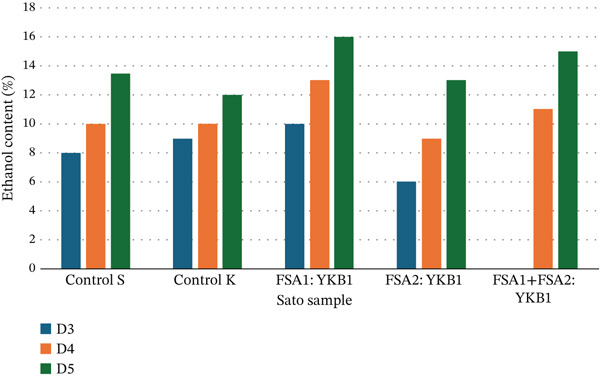
Ethanol content of Sato during fermentation.

An analysis of ethanol content variations during the 6‐day fermentation of Sato revealed a consistent increase in ethanol concentration across all fermentation samples. Ethanol levels exhibited a continuous upward trend from Day 0 to Day 6. Among the samples, Sato fermented with the third starter culture (FSA1:YKB1) achieved the highest ethanol concentration, reaching 16.0 ± 0.01*%* (Figure 4).

### 3.8. Yeast DNA Sequencing

**Figure 5:**
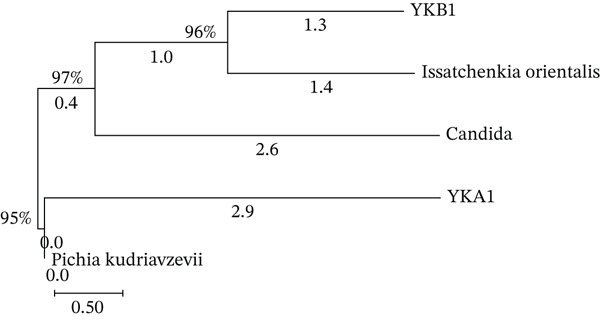
Phylogenetic tree of isolated YKA1 and YKB1 strains.

Both isolated yeast strains, YKA1 and YKB1, underwent DNA sequencing at Macrogen using NS1 and NS8 primers. The sequencing results identified YKA1 as having 86.74% similarity to *Pichia kudriavzevii* and YKB1 as having 95.57% similarity to *Issatchenkia orientalis* (Figure 5).

## 4. Discussions

The isolation and selection of fungi and yeast from traditional Loog‐pang fermentation starters highlight the diversity of microbial strains involved in starch degradation and alcohol fermentation. The identification of *P. kudriavzevii* and *I. orientalis* as dominant yeast species aligns with previous studies indicating their critical roles in saccharification and ethanol production in traditional rice wine fermentation [13, 16]. The high starch degradation efficiency observed in FSA1 and FSA2, as well as the superior sugar‐to‐alcohol conversion of YKB1, suggests that these strains could be optimized for controlled fermentation applications. Notably, YKB1 exhibited strong alcohol tolerance, a trait commonly associated with its ability to withstand stress during fermentation [17]. The identification of efficient starch‐degrading fungi and high ethanol‐producing yeasts is crucial for improving the efficiency of black glutinous rice wine production, ensuring consistency and enhanced alcohol yields [18].

The dynamic changes in TDS and pH levels throughout the fermentation process are consistent with the metabolic activity of yeast and fungal enzymes acting on rice starch and sugars. The initial rise in TDS during the first 3 days indicates active enzymatic breakdown of complex carbohydrates, while the subsequent decline reflects sugar consumption by yeast for ethanol production [18]. The pH decrease observed across all fermentation conditions is characteristic of organic acid accumulation, which is a natural outcome of yeast metabolism in rice wine fermentation [19, 20].

The highest ethanol yield of 16.0% recorded in the FSA1:YKB1 fermentation condition underscores the importance of strain selection in maximizing alcohol output, aligning with reports that *I. orientalis* contributes significantly to high ethanol production due to its robustness and efficiency in fermenting high‐sugar substrates [21]. This finding supports previous research advocating for the co‐inoculation of specific yeast and fungal strains to enhance fermentation [22].

The application of DNA sequencing confirmed the taxonomic identity of the selected yeast strains, providing a molecular basis for understanding their fermentation capabilities. The genetic similarity of YKB1 to *I. orientalis* further supports its suitability for industrial fermentation, given its known adaptability and ethanol tolerance in high‐sugar and low‐pH environments [23]. This study demonstrates that the selected fungal and yeast isolates are highly promising for improving traditional Loog‐pang Sato starter cultures, with potential applications in commercial rice wine fermentation. Future research should explore the genetic and enzymatic pathways contributing to their fermentation capabilities to optimize large‐scale production [24, 25]. Additionally, further characterization of the microbial interactions between fungal and yeast strains could provide deeper insights into synergistic fermentation processes and flavor development in traditional rice wines [26, 27].

## Funding

Funding for this study was provided by Roi Et Rajabhat University under Project Code 182033.

## Conflicts of Interest

The authors declare no conflicts of interest.

## Data Availability

The data that support the findings of this study are available from the corresponding author upon reasonable request.
